# Physical Properties of PDMS (Polydimethylsiloxane) Microfluidic Devices on Fluid Behaviors: Various Diameters and Shapes of Periodically-Embedded Microstructures

**DOI:** 10.3390/ma9100836

**Published:** 2016-10-15

**Authors:** Changhyun Roh, Jaewoong Lee, ChanKyu Kang

**Affiliations:** 1 Biotechnology Research Division, Advanced Radiation Technology Institute (ARTI), Korea Atomic Energy Research Institute (KAERI), 29 Geumgu-gil, Jeongeup, Jeonbuk 56212, Korea; chroh@kaeri.re.kr; 2 Department of Textile Engineering and Technology, Yeungnam University, 280 Daehak-Ro, Gyeongsan, Gyeongbuk 38541, Korea; jaewlee@yu.ac.kr; 3 Ministry of Employment and Labor, Major Industrial Accident Prevention Center, 34 Yeosusandallo, Yeosu-Si, Jeonnam 59631, Korea

**Keywords:** friction factor, PDMS bulging, embedded microstructures, hydraulic diameter, pressure drop

## Abstract

Deformable polydimethylsiloxane (PDMS) microfluidic devices embedded with three differently-shaped obstacles (hexagon, square, and triangle) were used to examine the significant challenge to classical fluid dynamics. The significant factors in determining a quasi-steady state value of flow velocity (*v*)*_QS_* and pressure drop per unit length (∆*P*/∆*x*)*_QS_* were dependent on the characteristic of embedded microstructures as well as the applied flow rates. The deviation from the theoretical considerations due to PDMS bulging investigated by the friction constant and the normalized friction factor revealed that the largest PDMS bulging observed in hexagonal obstacles had the smallest (∆*P*/∆*x*)*_QS_* ratios, whereas triangle obstacles exhibited the smallest PDMS bulging, but recorded the largest (∆*P*/∆*x*)*_QS_* ratios. However, the influence of (*v*)*_QS_* ratio on microstructures was not very significant in this study. The results were close to the predicted values even though some discrepancy may be due to the relatively mean bulging and experimental uncertainty. The influence of deformable PDMS microfluidic channels with various shapes of embedded microstructures was compared with the rigid microchannels. The significant deviation from the classical relation (i.e., *f~1/Re*) was also observed in hexagonal obstacles and strongly dependent on the channel geometry, the degree of PDMS deformation, and the shapes of the embedded microstructures.

## 1. Introduction

Microfluidic devices have attracted considerable attention for their actuated control of micro-scale fluid behaviors. This advantage makes microfluidic devices a preferable choice in the fields of biomedical assaying, chemical analysis, electronic technology, and drug delivery [1,2,3]. Successful applications in devices, such as micro heat exchangers, reactors, concentrators, mixers, and separators, are increasingly being realized [4,5,6,7]. With the advances in soft lithography processes, complex and dedicated structures in the microchannels are being used widely to mimic microorganisms in the human body or microfluidics-based chemotaxis, or to increase chemical mixing using different shapes of embedded structures [8,9,10]. The micro-mixer, or chemotaxis device, having complex embedded structures, has been used with moderate and shallow microchannels because of its simplicity [9,11]. Among several polymer materials used in microfluidic devices, PDMS (polydimethylsiloxane) has become the preferred material owing to its outstanding material properties [12,13].

The observation of fundamental flow behavior in microchannels has been preceded by the remarkable advancements in experimental and theoretical investigations. Therefore, the continuum assumption and use of the Navier-Stokes equations are generally assumed to be valid for liquid flows in microchannels and are experimentally in good agreement in many studies [14,15,16]. On the other hand, several studies using rigid microchannels reported significantly greater values of the friction constant (*fRe*) for the microchannels than for the classical channels [17,18]. The decreased channel aspect ratio (*α*) and the ratio of channel height (*h*) to width (*w*) in rectangular microchannels increased the friction factor significantly [19]. Several studies have suggested that the unexpectedly large discrepancies from the predicted values with respect to the friction factor (*f*) data were due to experimental uncertainties, microchannel geometry, and surface roughness [20]. Among these, surface roughness should be investigated carefully but considered as a less significant factor compared to other factors. Despite the surge in PDMS microchannel applications within complex structures, there have been limited studies on interactions between fluids and soft embedded microstructures, or to determine if the influence of shapes of embedded obstacles is a significant factor in the flow behaviors with PDMS soft materials.

Deformation of microfluidic channels fabricated from soft PDMS changes flow behavior and can sometimes lead to ambiguous results. Gervais et al. [21] examined PDMS microfluidic channel deformation under various flow rates and found that microchannel bulging was greatest at the inlet (highest pressure) and least at the outlet. It was found that when the flow rates were doubled, the measured pressure drop increased by only approximately 55%, rather than a factor of two as would be expected with a rigid microfluidic system. Hardy et al. [22] investigated the microchannel bulging effects using florescence microscopy with Rhodamine 6G dye and reported a 65% increase in pressure drop when the flow rates were doubled due to bulging of the PDMS microchannels. Similarly, in situ pressure measurements with deformable PDMS microfluidic channels showed that measured pressure drops were smaller than the values predicted by rigid channel theories [23]. Sollier et al. [24] used various polymer materials, such as Thermoset Polyester (TPE), Polyurethane Methacrylate (PUMA), and NorlandAdhesive 81 (NOA81), to investigate the flow behaviors in microfluidics channels, which were strongly affected by microchannel materials. This study examined whether the influence of the material property is a significant factor on the flow behaviors.

In the previous study, increasing microfluidic channel height provided more reliable results when pressure-driven flows moved through PDMS microfluidic channel with circular embedded obstacles [25]. The present article reports the flow behavior over long, shallow PDMS microfluidic channels containing periodically spaced obstacles of three different shapes (i.e., hexagon, square, and triangle) and diameters (i.e., 132, 152, 172 µm), because embedded obstacles with various shapes in PDMS microchannels have been widely used in many experimental applications, such as micro-mixers and 3D cell culture systems due to their superior flow mixing and miniaturized human organs [26,27]. In addition, despite the outstanding performance, the interaction between flow and microstructure interaction flow is not clearly understood. This study identifies the influence of the microstructure on the flow property under a quasi-steady state. The pressure was measured experimentally using a pressure transducer, and the flow velocity was determined using a charge-coupled device (CCD) camera to quantify the flow characteristic features by determining the Reynolds number (Re), friction constant (*fRe*), and normalized frictional constant (C*). The experimentally determined flow characteristics were then compared with classical theories to identify the PDMS channel bulging phenomenon with 1 µL/min, 2 µL/min and 4 µL/min flow rates. In addition, the degrees of deviation from classical theory due to PDMS channel bulging were analyzed with embedded shapes and diameters. The significant effect deformable microchannels have on the flow behavior provides useful information for the design and fabrication of microfluidic-based lab-on-a-chip systems.

## 2. Analysis of Flow and Theory

To calculate the flow parameters, the mean porosity was evaluated simply as the ratio of the channel volume available for flow to the total channel volume. In the present experiments, the hydraulic diameter obviously varied periodically along the microfluidic channel length due to the presence of the obstacles. The hydraulic diameter without obstacles was 28.26 µm. Therefore, the mean hydraulic diameter for the obstacle-laden microchannels was calculated as follows:

(1) $$\bar{D_{H}}=4\frac{Cross\;\sec tional\;area\;\times L}{Wetted\;perimeter\times L}=4\frac{Channel\;volume\;available\;for\;flow}{Total\;wetted\;channel\;surface\;area}$$

The numerator term, which is the channel volume available for flow, is the volume of embedded obstacles subtracted from the volume of the rectangular microchannels. The denominator term, which is the total wetted channels surface area, was considered the surface areas of rectangular microchannels and wetting obstacles when the flow moved through the microchannels.

The experimental Reynolds number was calculated using the mean hydraulic diameter $\bar{D_{H}}$, the mean quasi-steady fluid velocity $V_{QS}$ (as determined from the optical measurements of the liquid front and discussed further below), fluid density *ρ*, and dynamic viscosity *µ*. Thus

(2) Re=ρVQSDH¯μ

The total measured pressure drop in the microfluidic system during each experiment, P_sys_, was calculated using Equation (3) [28]

P_system_ = ∆P_tubing_ + ∆P_needle_ + ∆P_entrance_ + ∆P_obstacles_ + ∆P_cap_ + ∆P_exit_ (3)

where ∆P_tubing_ is from the losses in the connecting tubing/fittings, ∆P_needle_ is due to loss in the chip injection needle, ∆P_entrance_ is the loss due to expansion at the microfluidic channel entrance, ∆P_obstacles_ represents the losses due to the obstacles encountered by the flow along the microchannel, ∆P_cap_ is the pressure drop due to capillarity effects at the free surface front during the start-up flow, and ∆P_exit_ is the pressure drop in the air exiting the microchannel in front of the liquid flow. The total pressure loss by the tubing and exit was less than 0.5% of the actual experimentally measured pressure drops, and needle and entrance effects were eliminated from the measured values of the increasing pressure data, as discussed below. The capillarity effect, ∆P_cap_, at the liquid-free surface was not negligible in this study, and this effect was managed carefully during an analysis of the first-order response system.

The pressure drop across the microchannel length *L* in steady-state laminar flow is defined as [23]

(4) $$\Delta p=Q\frac{12\mu L}{h^{3}w}=v\frac{12\mu L^{2}}{h^{3}}$$

where ∆*P* is the pressure drop along microchannel length *L*, *Q* is the imposed flow rate, *h* is the microchannel height, *v* is flow velocity, and *µ* is the viscosity of the fluid. Because the flow rate, *Q*, is a function of the flow velocity and area, Equation (4) can be rearranged as a function of the flow velocity.

The transient start-up response of the microfluidic system was analyzed as a simple first-order system by several researchers [29,30,31]. After starting from zero, the velocity increased asymptotically towards a quasi-steady state value (*v_QS_*) related to the flow rate imposed by the syringe pump. The term, a quasi-steady state flow, is mentioned frequently in the application of microfluidic devices due to the insufficient length of microchannels [32]. Therefore, for a first-order system

(5) $$v(x)=v_{QS}[1-\exp(-\frac{x}{x_{DV}})]$$

The velocity decay constant *X_DV_* exhibited by such a system is due to the combined effects of liquid inertia, viscous friction and other losses, meniscus capillarity, PDMS bulging, and syringe pump response. The associated pressure drop per unit length of fluid-filled channel, ΔP/Δx, was also analyzed as a simple first-order system [33]. Although the measured system pressure increased as the fluid filled the microfluidic channel, the pressure drop per unit length of fluid-filled channel $\left(\frac{\Delta P}{\Delta x}\right)$ decreased asymptotically towards a quasi-steady state value ${\left(\frac{\Delta P}{\Delta x}\right)}_{QS}$ as the fluid accelerated towards *V_QS_*. Thus

(6) $$\frac{\frac{\Delta P(x)}{\Delta x}(x)-{\left(\frac{\Delta P}{\Delta x}\right)}_{QS}}{{\left(\frac{\Delta P}{\Delta x}\right)}_{i}-{\left(\frac{\Delta P}{\Delta x}\right)}_{QS}}=\exp(-\frac{x}{x_{DP}})$$

where ${\left(\frac{\Delta P}{\Delta x}\right)}_{i}$ is an initial value of pressure drop per unit length. The first-order system decay constant associated with the pressure *x_DP_* in such a system is, as before, due to the combined effects of liquid inertia, viscous friction and other losses, meniscus capillarity, PDMS bulging, and the syringe pump response. Analysis and fitting of the velocity and pressure drop data to Equations (5) and (6) were performed using Origin software (OriginLab, Northhampton, MA, USA).

The Darcy friction factor *f* is often used to characterize the viscous losses in microfluidic systems and is defined as

(7) $$f=\bar{D_{H}}{\left(\frac{\Delta P}{\Delta x}\right)}_{QS}\frac{1}{\rho {\left(\bar{v}\right)}_{QS}^{2}/2}$$

where ${\left(\Delta P/\Delta x\right)}_{QS}$ is the quasi-steady state pressure drop per unit channel length. The friction constant (*fRe*) for rectangular channels is a function of *α* and can be expressed in the form of a polynomial equation. The relationship is expressed as [18]

*fRe* = 96(1 − 1.3553*α* + 1.9467*α*^2^ + 1.7012*α*^3^ + 0.9564*α*^4^ − 0.2537*α*^5^) (8)

Equation (8) can be useful for microfluidic channels with embedded obstacles when the flow reaches the steady-state flow. The measured data can often be reported as a normalized friction constant (C*), which is used widely to observe a discrepancy from the laminar theory. The value of C* is a non-dimensional number using the theoretical value of *fRe* and is defined as [20]

(9) C*=fReexperimentfRetheory

where *fRe*_experiment_ is the experimentally determined value and *fRe_theoretical_* is the theoretical value determined by the channel geometry, as shown in Equation (9). The used value of *α* in this experiment was 0.06.

Careful consideration of the various sources of experimental uncertainties is a prerequisite for a reliable interpretation of the experimental results of the pressure drops and friction factors in the microchannels. Two basic types of uncertainties, systematic and random uncertainties, were analyzed including faults in the measuring instrument and in the technique itself. The definition of the microfluidic friction factor yields the fractional uncertainty in the measurements of the friction factor *f* as follows [34]:

(10) $$\frac{U_{f}}{f}={\left[{\left(\frac{U_{{\bar{D}}_{H}}}{{\bar{D}}_{H}}\right)}^{2}+{\left(\frac{U_{\Delta P}}{\Delta P}\right)}^{2}+{\left(\frac{U_{L}}{L}\right)}^{2}+{\left(\frac{U_{\rho }}{\rho }\right)}^{2}+{\left(\frac{2U_{\bar{v}}}{\bar{v}}\right)}^{2}\right]}^{1/2}$$

The term *U* is an acronym for the uncertainty, *ρ* is the fluid density, and $\bar{v}$ is the mean fluid velocity.

## 3. Results and Discussion

**Figure 1:**
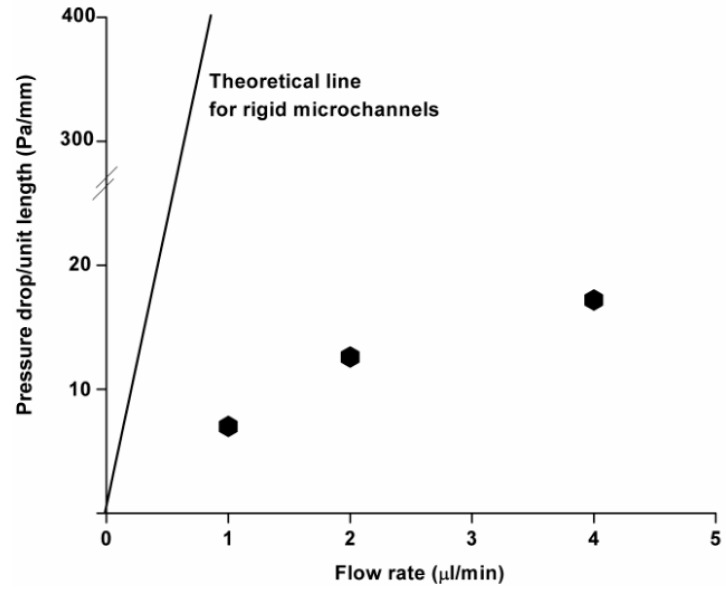
Experimentally and theoretically determined relationship between the channel pressure drop and imposed flow rate (FH4).

Equation (4) indicates that a linear relationship between the imposed flows and the pressure drop that developed along the channel can be observed in the microfluidic channels. On the other hand, pressure-induced deformation of the microfluidic channels fabricated from flexible PDMS can lead to non-linear behavior between the imposed flows and the pressure drop as a result of uncontrolled microfluidic channel expansion, as shown in Figure 1.

This suggests that pressure-driven flow has a tendency to deform shallow PDMS microchannels despite the embedded obstacles and low flow rates, which could contribute to less PDMS bulging. An analysis of the bulging data, as shown in Table 1, shows direct evidence of the pressure induced bulging of three different shapes of obstacles and shows an abrupt change in area in the microfluidic channel. The maximum bulging ratio is observed in cases where the hexagonal obstacles are embedded in the microfludic channels. The proper selection of microfluidic devices with various shapes of embedded obstacles is required to minimize the experimental discrepancy from the predicted values. As expected, low flow rates induced minor PDMS bulging with the smallest mean as-fabricated hydraulic diameter in the microchannel. On the other hand, the increase in hydraulic diameter due to PDMS bulging was less than 6%, even though the applied flow rate doubled.

**Figure 2:**
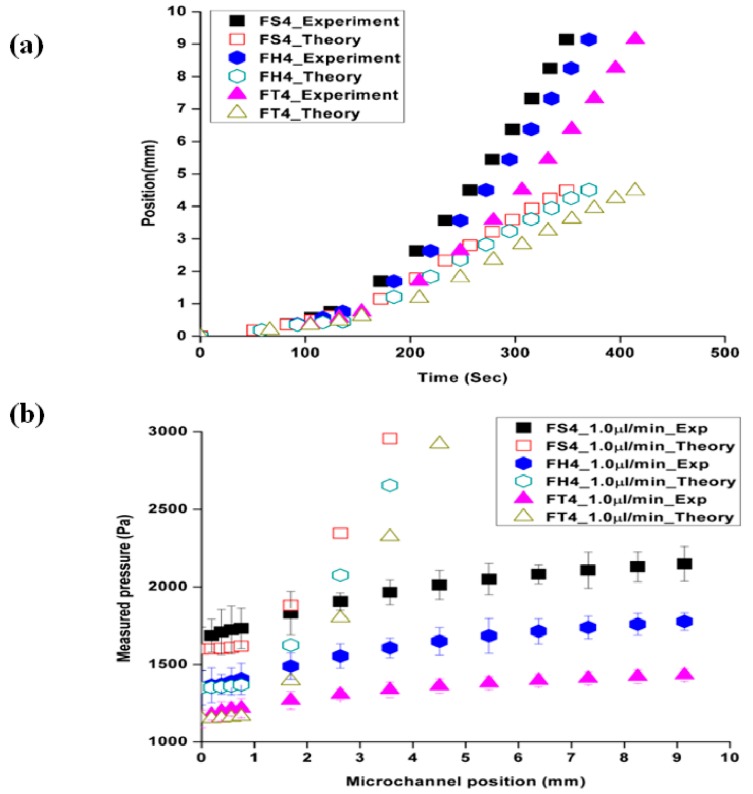
Typical data utilized for the flow analyses in the present work. Data shown are for flow through channels with three different shapes of obstacles at an imposed flow rate of 1 μL/min. (**a**) comparison of the experimental and theoretical position as a function of time and (**b**) developed pressure data as a function of the position.

Figure 2a presents the flow velocity, which is captured by the CCD camera and recorded in an optically tracked movie file, as a function of microchannel positions and times as the fluid front reached each obstacle along the channel. The difference with micro-particle tracking velocimetry techniques was less than 7% ± 2%. Figure 2b presents the pressure data for the three different shapes of obstacles as a function of the position. The experimentally achieved data was compared with Equation (4) to identify the flow velocity and pressure changes; this indicates that the observed deviation of experimental results from the theoretical prediction was due to the PDMS bulging effect. As a result, it is assumed that the material, particularly soft material, can contribute to the fluid dynamics. An analysis of the transient response of the microfluidic system during these start-up flow experiments was explored more closely using Equations (5) and (6).

**Figure 3:**
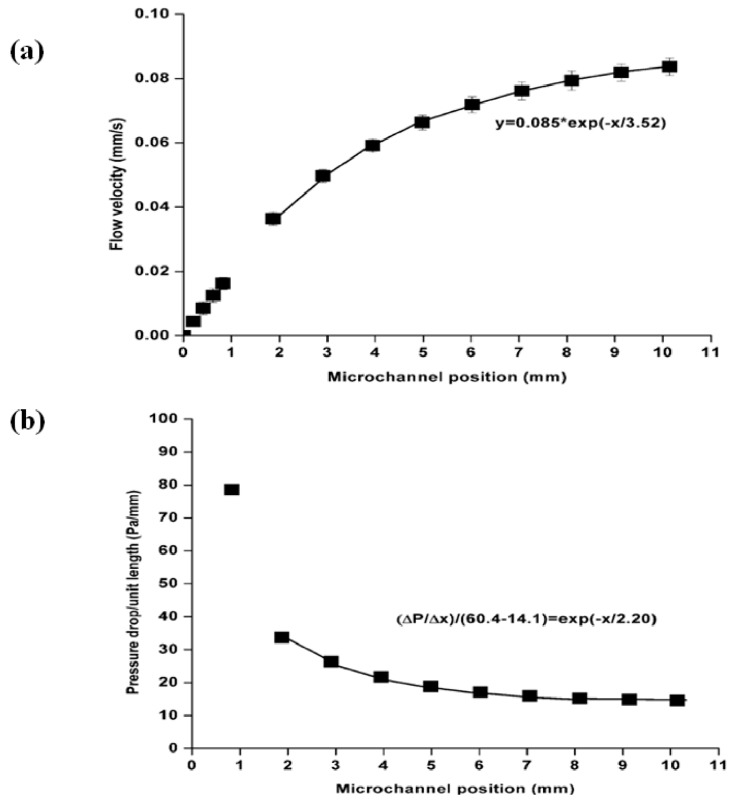
Typical derived data and curve fits for shallow microchannels with an obstacle FS2 at a flow rate of 1.0 μL/min (**a**) meniscus velocity and (**b**) inlet pressure.

The applications of these two equations are applied in Figure 3 and are summarized in Table 2. The curve fits shown in Figure 3 were only applied to the data after the 2 mm position at the end of the microchannel to avoid the entrance effect transients during the initial start-up. The same procedure was applied to all the experimental data in this investigation. The meniscus velocity increased toward a quasi-steady constant value, *V_QS_*, and ∆*P*/∆*x* decreased toward a corresponding quasi-steady value (∆*P*/∆*x*)*_QS_*. The ratios of $\frac{v_{QS}(2.0\mu l/\min)}{v_{QS}(1.0\mu l/\min)}$ for all obstacle diameters were almost double in Table 2, which suggests that the measured values of *V_QS_* doubled as the imposed flow rate from the syringe pump doubled.

The value of (Δ*P*/Δ*x*)*_QS_* is a function of the microchannel hydraulic diameter and flow rate, as expected. This means that as the applied flow rates doubled, the observed pressure drop also doubled, as shown in Equation (4). On the other hand, the ratios of $\frac{{\left(\Delta P/\Delta x\right)}_{QS}(2.0\mu l/\min)}{{\left(\Delta P/\Delta x\right)}_{QS}(1.0\mu l/\min)}$ for all cases in this study suggested that doubling the imposed flow rate did not double (Δ*P*/Δ*x*)*_QS_* for the shallow microfluidic channels, even though it was improved in the case where the obstacles had small mean hydraulic diameters along with increasing flow rate at 4.0 µL/min, presumably due to the PDMS bulging effects. This suggests that PDMS deformation is dependent on the shape of embedded obstacles and has a more significant influence on (Δ*P*/Δ*x*)*_QS_* in this study than *V_QS_*. The largest effect in this study was observed in hexagonal obstacles. The coefficients of determination (*R*^2^) for the best fits varied from 0.937 < *R*^2^ < 0.998 for *V* and from 0.935 < *R*^2^ < 0.998 for ∆*P*/∆*x*. The determined values from Table 2 were used to analyze the friction constant for the 1.0 µL/min, 2.0 µL/min and 4.0 µL/min flow rates. In general, as the fluid velocity reached a constant value, the slope of the pressure curve for these experimental conditions also reached a constant value simultaneously. On the other hand, the exponential curve fit to the velocity data of Figure 3 identified a decay constant (*X_DV_* = 3.52 mm) that was significantly larger than the decay constant found for the pressure drop data for the same experiment (*X_DP_* = 2.20 mm). Table 3 lists the decay constants in the first-order response for shallow microchannels. The system pressure would tend to equalize faster than the liquid could accelerate towards its quasi-steady state velocity in the microchannel.

**Figure 4:**
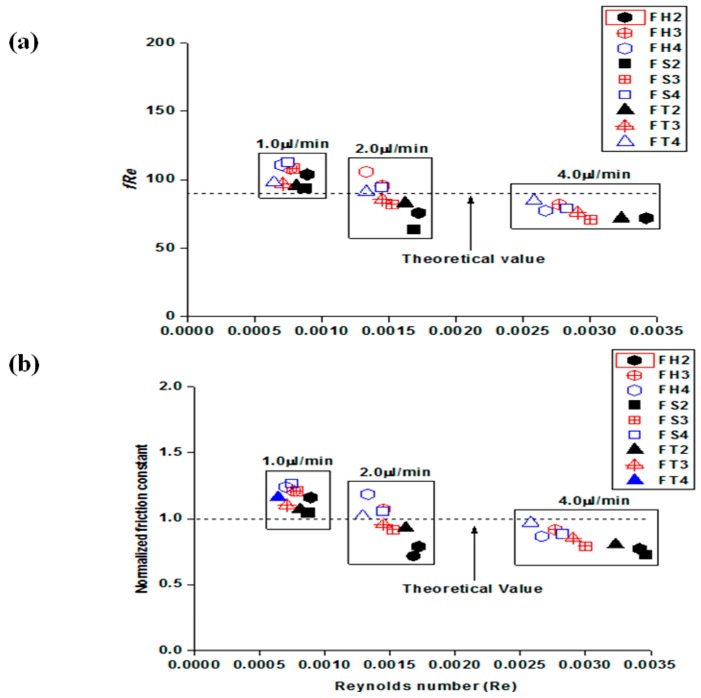
(**a**) Friction constants of three different shapes of obstacles at three different flow rates and (**b**) the normalized friction constants as a function of a low Reynolds number with three different shapes of obstacles.

The relationship between the friction constant (*fRe*) and low Reynolds number, which was obtained for liquid flows through three different shapes of embedded obstacles in the microchannels, is represented by the theoretical values in Figure 4a. These results suggest that increasing the applied flow rate decreases the friction constant. In addition, the usual laminar flow trend of decreasing the friction constant with increasing Reynolds number (Re) is evident, even though the range of Reynolds number (Re) investigated was small. The different sizes of the embedded obstacles generated different characteristic features. The decreasing mean hydraulic diameter allowed the liquid to move quickly through microfluidic channels which eventually induced an increase in pressure drop and PDMS bulging. Figure 4b shows the normalized friction constant (C*), which is expressed as a function of Re to identify the significant experimental deviations from the macroscale laminar flow predictions at the same Re. As displayed in Figure 4b, there were small deviations observed in all flow rates investigated (i.e., 1.0 µL/min and 2.0 µL/min). An additional investigation with 4.0 µL/min also produced values closer to the predicted values but there were small deviations regardless of the shapes of embedded obstacles. The value of C* ranged from 0.72 to 1.18 in the case of 2.0 µL/min, in which the mean value is close to the desired value (C* = 1.0). An increase in flow rate to 4.0 µL/min showed that the value of C* ranged from 0.71 to 0.96.

Even though increasing flow rate generates a reduced friction constant, the small deviations observed in all flow rates have several explanations. One is that theoretical PDMS deformation in 1.0 µL/min should be close to half that of 2.0 µL/min and a quarter that of 4.0 µL/min which contributes to the large deviation in comparison to high flow rates. On the other hand, a relatively large deviation was observed in Table 1; the ratio of PDMS bulging in 1.0 µL/min was much larger than the expected values compared to 2.0 µL/min and 4.0 µL/min. Another explanation is the large experimental uncertainty introduced at the beginning of this paper and shown in Table 4. The average experimental uncertainties for Re and *f* were 7.27% and 9.16%, respectively, at 1.0 µL/min, which is larger than 2.0 µL/min (i.e., 4.83, 8.42) and 4.0 µL/min (i.e., 4.79, 7.42). The investigated microfluidic channel height was shallow and had low flow rates, which generally contributes to the experimental uncertainties. The experimental uncertainties could be due to the large deviations from the predicted values, but the investigation into the effects of the experimental uncertainty was limited within this study. A combination of the two factors may have contributed to the large deviations from theoretical expectations. The expanded PDMS microfluidic channels eventually reduced the flow velocity along with a pressure drop. As a result, the flow property changes in flexible PDMS microfluidic channels leads to significant changes in both the friction constant and normalized friction constant. This phenomenon induced significant deviations from the theoretical values in flexible microchannels.

**Figure 5:**
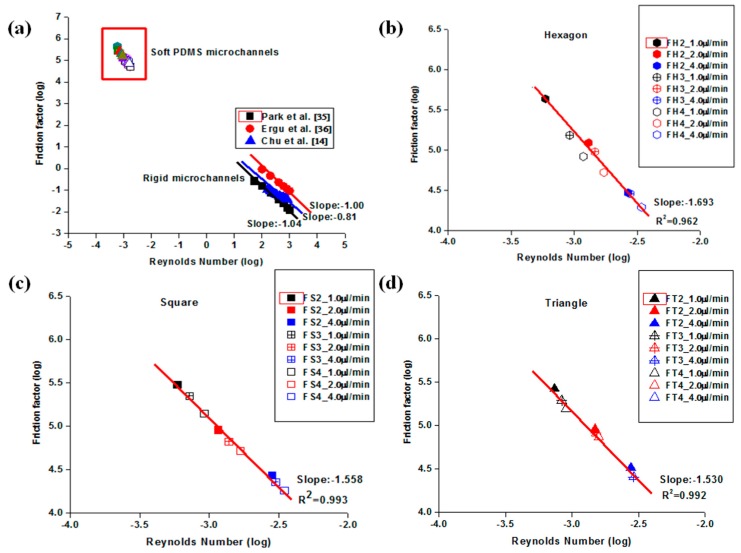
Friction factor as a function of low Reynolds number: (**a**) comparison of this study with rigid microchannel studies; (**b**) analysis of hexagonal obstacles; (**c**) analysis of square obstacles; and (**d**) analysis of triangular obstacles.

Figure 5 shows the influence of the three different shapes of embedded obstacles compared to rigid microchannel studies. The microchannel made from rigid materials (i.e., silicon and acryl) was shown to be in relatively good agreement with the predicted values (i.e., *f~Re*^−1^), whereas the slope became much steeper than −1 in all cases. The most significant deviation from the theoretical prediction was observed in hexagonal obstacles, which showed the most bulging, whereas the smallest deviation was observed in the triangular obstacles, which had the least bulging in this study. The deviation from the theoretical prediction could be related to the degree of PDMS bulging. This information provides a protocol to select both the appropriate obstacle and microchannel geometry in future applications.

## 4. Materials and Methods

### 4.1. Materials

A commercially available food dye solution was procured from ESCO Foods Inc. (San Francisco, CA, USA). A PDMS RTV 615 silicon elastomeric kit from MG Chemicals (Burlington, ON, Canada) was used. A mixing ratio of 10:1 (elastomer: cross-linker) was used. The density and viscosity of the food dye were measured at room temperature using a tensiometer (KSV Sigma 702, Dyne Testing Ltd., Staffordshire, UK) and viscometer (ViscoLiner 800, Galvanic Applied Sci., Lowell, MA, USA), respectively.

### 4.2. Microfluidic Chip Fabrication

Each microfluidic chip was fabricated by standard photoresist-based soft lithography. AutoCAD software (AutoDesk Inc., San Rafel, CA, USA) was used to produce a mask design that was then printed on a transparent film by CAD/Art Service Inc. (Bandon, OR, USA). A positive photoresist (AZ P4620) was applied to a 4-inch silicon wafer by Silicon Quest International Inc. (Santa Clara, CA, USA). A mold of height ~5 mm was placed on the wafer surrounding the photoresist layer, and the mixed PDMS compound was then poured onto the wafer inside the mold structure to produce a 5 mm-thick chip of the fluidic structure to be characterized. PDMS was also poured directly onto a bare silicon wafer and cured at 80 °C for 1 h to produce a control base layer. The fluidic chip layer was peeled off the wafer/photoresist and holes for the inlet and the outlet ports were fabricated using a 19-gauge punch (Technical Innovations Inc., Angleton, TX, USA). A plasma cleaner was used to remove the impurities and contaminants on the surface of the fluidic layer and glass slide, and the two layers were then aligned carefully for a final thermal bonding treatment in an 80 °C oven for 18 h. Additional plasma treatment was applied to the sealed microfluidic channels to maintain strong hydrophilicity [37]. The dimensions of the fabricated microfluidic chips were a measured width of 243 ± 1 μm, a measured height of 15 ± 1 μm and a measured wall thickness of 5 ± 1 mm. A row of aligned periodic obstacles was arranged along the centerline of each channel. The obstacle shapes investigated included squares, hexagons, and equilateral triangles. The micro-fabricated obstacles in this study exhibited the characteristic lengths of 132 μm, 152 μm, and 172 μm which were fabricated with low aspect ratio microchannels (*α* = 0.062). In this study, unused microfluidic channels were used each time and the process was repeated a minimum of three times. Three characteristic dimensions, 132 μm, 152 μm, and 172 μm, were fabricated with low aspect ratio microchannels (*α* = 0.062).

### 4.3. Experimental Procedure

**Figure 6:**
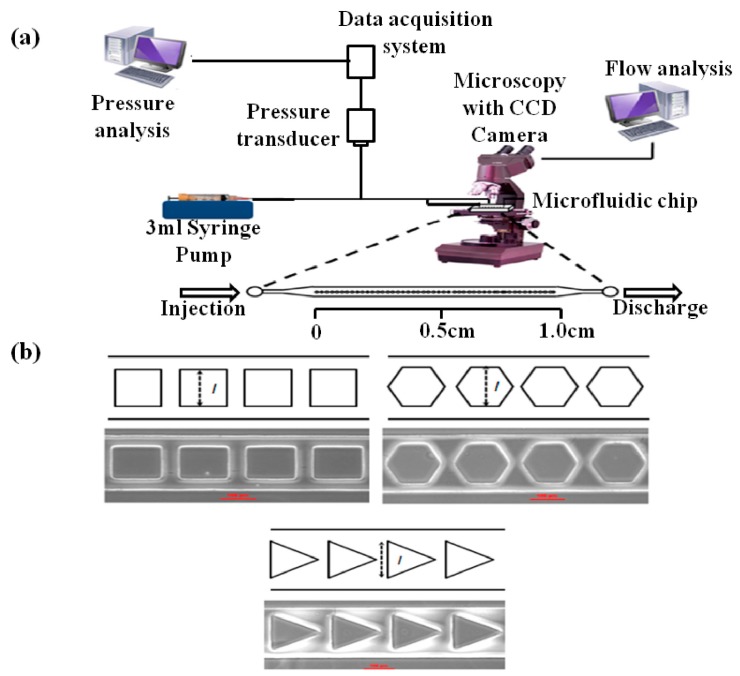
(**a**) Schematic diagram of the experimental apparatus; and (**b**) close-up image of the obstacles.

A commercially available liquid food dye solution was used for the experimental investigations of the flow properties. The liquid properties were analyzed before the experiment because food dye solutions are used frequently to visualize PDMS microfluidic channels using transmitted light microscopy [38]. The density of liquid solution was measured to be 0.6848 ± 0.006 g/cm^3^, while the viscosity was determined to be 1.5625 ± 0.0016 cP. The commercial liquid food dye used in this study had a lower density than water and had a similar property to other commercial food dyes (i.e., McCormick & Company Inc., Baltimore, MA, USA). In addition, the viscosity of flow was constant throughout the microfluidic channels because the measured temperature changes inside the microfluidic devices showed a difference of less than 0.1 °C, which was in agreement with the reference study [39]. Therefore, the properties of the fluid were assumed to be constant at room-temperature, which did not influence the change in viscosity and density. Figure 6a presents a schematic diagram of experimental setup and Figure 6b shows the obstacles used in the microfluidic chip. Tygon tubing connected a syringe pump to the microfluidic chip at the microfluidic chip inlet. The flow rates were maintained constant by the syringe pump (+0.5% accuracy) at either 1.0 μL/min or 2.0 μL/min. The transient position of the flow front along the microchannel was followed by microscopic observations (Nikon A1, Melville, NY, USA) and recorded with an image-intensifying CCD camera (Nikon Digital Sight DS-Q1MC, Nikon, Seoul, Korea). The recorded image movie files enabled a direct determination of the position of the liquid front as a function of time and as a calculation of the velocity of the liquid front versus time. The position of the liquid front was analyzed using Nikon viewer software. This method was compared with micro-particle tracking velocimetry techniques for its reliability [40]. The image of PDMS deformation observed by Nikon A1 confocal microscopy was analyzed by Imaris 7.2 (Bitplane Inc., Concord, MA, USA) after the filtering and rendering processes, which follows the same method reported in [21]. The time-dependent applied pressure was measured directly throughout each experiment using a gauge pressure transducer (PX138-0.3D5V, Omega Engineering Inc., Swedesboro, NJ, USA). The transducer voltage was digitized and recorded with a computerized data acquisition system (DI-148U, DATAQ Instrument, Akron, OH, USA). The pressure data, which was recorded on computer 1 (see Figure 6a) by time stamping the pressure data file at the moment when the liquid front just touched the first obstacle, were synchronized in time with the CCD image movie files recorded using computer 2 (see Figure 6a). The obstacle shapes and characteristic lengths are defined in Figure 6b.

**Figure 7:**
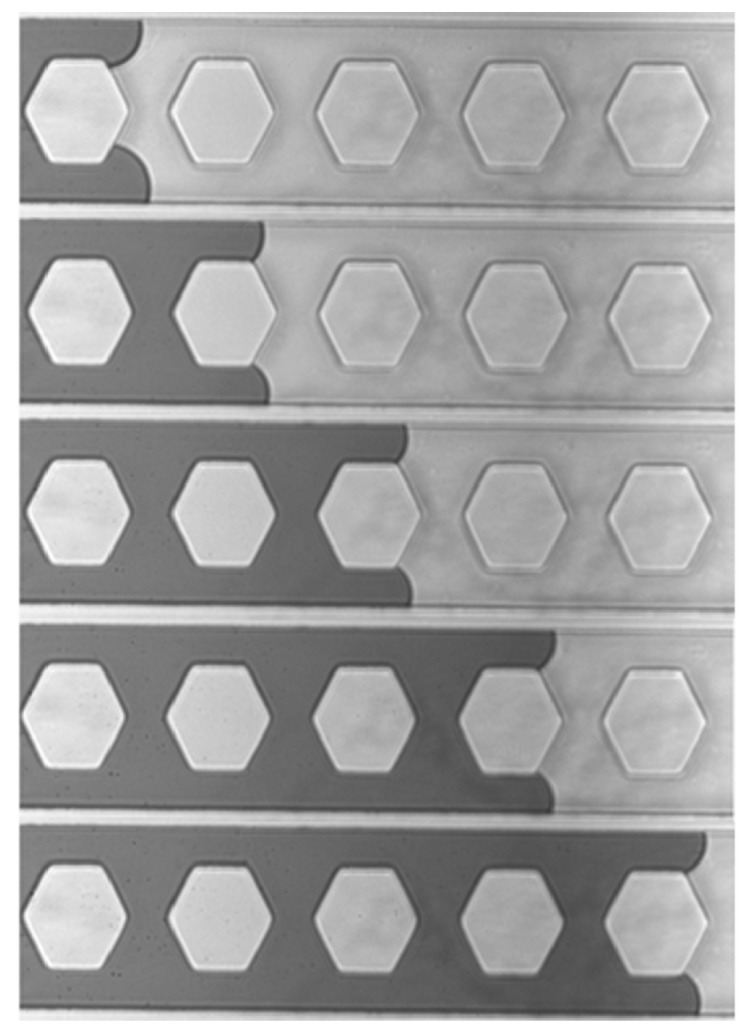
Flow images recorded for the hexagonal obstacle in the microfluidic channel (FH4, 1.0 μL/min): t = 123.5 s (top); t = 126.21 s; t = 128.92 s; t = 131.63 s; t = 134.34 s; and t = 137.04 s (bottom).

Figure 7 shows the typical results in a series of images of the fluid front as it moves through a channel with hexagonal obstacles without bubble formation. To reduce the spatial variation, the flow velocity was measured, where both sides of the flow are simultaneously touching the obstacle before the flow passes through the obstacle. The measured spatial variation is less than 20 ± 3 μm. This experiment does not deal with the effects of the surface roughness because the average roughness featured in this study was small (<20 nm) and operated at very low Reynolds numbers (10^−4^~10^−3^) in the microchannels. The main target of the flow rates was 1 μL/min and 2 μL/min but an additional flow rate (4 μL/min) was investigated for three structures to determine the tendency of an increasing discrepancy at low flow rates.

## 5. Conclusions

This paper reports an experimental study into the flow velocities, pressure drops, friction constants, and associated normalized friction constants for very low Reynolds number flows through shallow rectangular PDMS microchannels with three different shapes of periodic obstacles. This study showed that the ignorance of PDMS deformation in shallow microfluidic channels due to the interaction between fluid flow and various shapes of microstructures is a critical factor in trusting the experimental accuracy in future applications, and has a considerable influence on the fluid dynamics. Many studies focused on high flow rate operations in PDMS microfluidic devices, in which severe PDMS bulging was easily anticipated. This study showed that the operation of microfluidic devices with low flow rates also induces unexpected deviations from the predicted values when PDMS microfluidic devices themselves have shallow depths. In addition, this study found that the selection of the embedded microstructures plays a significant role in controlling the degree of PDMS bulging and flow property, especially (∆*P*/∆*x*)*_QS_*.

The experimental analysis showed small deviations from classical theory. The experimentally determined friction constants for these low velocity flows with flexible shallow microfluidic channels have different characteristic features, which are dependent on the applied flow rates, mean hydraulic diameters, and shapes of the embedded obstacles. The experimental data showed a large friction constant at a lower flow rate (e.g., 1.0 µL/min), while another exhibited a lower friction constant compared to the predicted values (e.g., 2.0 µL/min and 4.0 µL/min). This trend extended to the normalized friction factor. This is possibly related to the relatively large average bulging ratio along with a large experimental uncertainty compared to 2.0 µL/min and 4.0 µL/min.

Shallow PDMS microfluidic channel with microstructures had a greater deleterious effect on the (∆*P*/∆*x*)*_QS_* values than on *V_QS_* for these experiments. This trend was significantly observed in hexagonal obstacles in comparison to that of square and triangle obstacles. This will become more plausible when this study is compared with rigid microchannels from other studies. An analysis of a rigid microchannel shows relatively good agreement with the theoretical prediction and matches the experimental observations, in which triangular-embedded microchannels exhibited the smallest bulging. It was obvious that the different shapes of embedded microstructures also exhibited different characteristics compared to that of rigid channels, due to the flow and soft structure interaction. The hexagonal obstacles in the microchannels exhibited the largest deviation (*f* ∞ *Re*^−1.693^) from the theoretical prediction of *f~Re*^−1^. The smallest deviation (*f~Re*^−1.530^) was observed with triangular obstacles in the microchannels and had the least bulging. These results suggest that the validity of the law of hydrodynamics on flexible PDMS microfluidic channels could be strongly dependent on the embedded microstructures which induced different microchannel deformation. The observed fractional uncertainty in the experimental Reynolds numbers was less than 9% for both flow rates, while the fractional uncertainty in the friction factor was less than 12% for both flow rates. Therefore, these results suggest that the effects of experimental uncertainty may contribute to a misinterpretation of the experimental results. Throughout this study, appropriate selection of the microfluidic channel geometry, flow rate, and embedded microstructures restricted PDMS bulging and provided reliable results during the PDMS microfluidic device operation.

## Figures and Tables

**Table 1:** Summary of channel hydraulic diameter change by flexible polydimethylsiloxane (PDMS).

Sample Designation	Mean As—Fabricated Hydraulic Diameter (μm)	Imposed Flow Rate					
		1.0 μL/min		2.0 μL/min		4.0 μL/min	
		Mean Bulging Modified Hydraulic Diameter (μm)	Ratio of Bulging (%)	Mean Bulging Modified Hydraulic Diameter (μm)	Ratio of Bulging (%)	Mean Bulging Modified Hydraulic Diameter (μm)	Ratio of Bulging (%)
FH2 (Hexagon)	23.8	25.7	7.98	25.8	8.40	26.4	10.92
FH3 (Hexagon)	24.6	26.4	7.32	26.7	8.54	27.1	10.16
FH4 (Hexagon)	25.2	27.0	7.14	27.1	7.54	27.5	9.13
FS2 (Square)	21.4	23.2	8.41	23.3	8.88	23.7	10.75
FS3 (Square)	22.9	24.7	7.86	24.9	8.73	25.2	10.04
FS4 (Square)	24.1	25.6	6.22	25.9	7.47	26.1	8.30
FT2 (Triangle)	24.9	26.4	6.02	27.1	8.84	27.4	10.04
FT3 (Triangle)	25.3	26.5	4.74	27.2	7.51	27.6	9.09
FT4 (Triangle)	25.7	26.6	3.50	27.3	6.23	27.9	8.56

**Table 2:** Analysis of the first-order response for flow velocity and pressure drop/unit length.

	Flow Velocity *v_QS_* (mm/s)					(∆P/∆x)*_QS_* (Pa/mm)				
	1.0 μL/min	2.0 μL/min	4.0 μL/min	*V_QS_* Ratio (a)	*V_QS_* Ratio (b)	1.0 μL/min	2.0 μL/min	4.0 μL/min	(∆P/∆x)*_QS_* Ratio (a)	(∆P/∆x)*_QS_* Ratio (b)
FH2	0.079	0.152	0.295	1.92	1.94	9.7	13.5	22.1	1.39	1.64
FH3	0.066	0.124	0.233	1.88	1.88	8.1	13.0	19.8	1.60	1.52
FH4	0.059	0.112	0.221	1.90	1.97	7.0	12.6	17.2	1.80	1.37
FS2	0.085	0.165	0.319	1.94	1.99	11.6	15.2	25.9	1.31	1.70
FS3	0.072	0.139	0.269	1.93	1.96	10.1	14.4	22.4	1.42	1.56
FS4	0.066	0.127	0.246	1.92	1.97	8.9	14.0	21.8	1.57	1.56
FT2	0.070	0.136	0.269	1.94	1.98	7.49	12.0	19.5	1.60	1.63
FT3	0.061	0.122	0.240	2.00	1.97	6.57	11.0	18.2	1.67	1.65
FT4	0.055	0.108	0.211	1.96	1.95	5.94	10.3	17.9	1.73	1.71

(a) The ratio between 1.0 μL/min and 2.0 μL/min; (b) The ratio between 2.0 μL/min and 4.0 μL/min.

**Table 3:** Analysis of the decay constants in the first-order response for shallow microchannels.

	Flow Velocity *v_QS_*			(∆P/∆x)*_QS_*		
	1.0 μL/min	2.0 μL/min	4.0 μL/min	1.0 μL/min	2.0 μL/min	4.0 μL/min
FH2	4.19	3.62	3.34	2.72	2.67	2.55
FH3	4.33	3.71	3.47	2.92	2.73	2.68
FH4	4.5	4.17	3.66	3.67	3.42	3.31
FS2	3.52	3.32	3.09	2.20	1.90	1.83
FS3	3.6	3.53	3.25	2.74	2.11	2.01
FS4	5.16	4.78	4.11	3.16	2.49	2.19
FT2	4.15	3.96	3.81	2.79	2.25	2.13
FT3	4.53	4.06	3.66	2.98	2.27	2.22
FT4	4.76	4.22	3.54	3.05	4.22	3.99

**Table 4:** Analysis of the fractional uncertainty in friction factor and Reynolds number.

	Fractional Uncertainty (%) in Reynolds Number			Fractional Uncertainty (%) in Friction Factor		
	1.0 μL/min	2.0 μL/min	4.0 μL/min	1.0 μL/min	2.0 μL/min	4.0 μL/min
FH2	8.99	5.16	6.89	8.94	9.26	8.11
FH3	8.17	4.30	5.66	11.19	7.39	7.01
FH4	6.31	4.69	4.55	8.19	6.40	6.88
FS2	8.45	5.52	6.68	8.81	8.58	7.43
FS3	6.14	4.79	5.04	9.20	9.62	8.39
FS4	6.40	4.85	4.74	7.78	8.26	7.34
FT2	6.99	4.88	4.66	10.76	8.99	8.01
FT3	7.99	4.62	4.58	10.40	6.87	6.23
FT4	6.03	4.64	4.33	7.23	10.45	7.41
